# The Economic Value of Environmental Services on Indigenous-Held Lands in Australia

**DOI:** 10.1371/journal.pone.0023154

**Published:** 2011-08-03

**Authors:** Kerstin K. Zander, Stephen T. Garnett

**Affiliations:** Research Institute for the Environment and Livelihoods, Charles Darwin University, Darwin, Northern Territory, Australia; University of Western Australia, Zimbabwe

## Abstract

Australians could be willing to pay from $878m to $2b per year for Indigenous people to provide environmental services. This is up to 50 times the amount currently invested by government. This result was derived from a nationwide survey that included a choice experiment in which 70% of the 927 respondents were willing to contribute to a conservation fund that directly pays Indigenous people to carry out conservation activities. Of these the highest values were found for benefits that are likely to improve biodiversity outcomes, carbon emission reductions and improved recreational values. Of the activities that could be undertaken to provide the services, feral animal control attracted the highest level of support followed by coastal surveillance, weed control and fire management. Respondents' decisions to pay were not greatly influenced by the additional social benefits that can arise for Indigenous people spending time on country and providing the services, although there was approval for reduced welfare payments that might arise.

## Introduction

The market-based Payments for Environmental Services (PES) is being held up as a means for reducing poverty in places rich in natural resources [1], [2]. In this paper we assess the willingness of Australian society to pay for environmental services that can be provided by Indigenous people on their land in northern Australia and evaluate the extent to which this is reflected by current levels of investment. We also investigate which services would attract greatest support and the reasons why some Australians would rather pay for none at all.

Land management jobs currently available to Indigenous people in many parts of northern Australia are among the few available in many communities where poverty underlies a 17 year gap in life expectancy, high levels of preventable diseases, and exceptionally low measures of social well-being [3]. Most Indigenous land management jobs are currently funded by Government as part of a national five year trial starting in 2008 and worth about US$40 million per year; others are supported by non-government organisations, businesses and philanthropic trusts. Payments for land management have particular benefit for Indigenous people since they allow fulfilment of cultural responsibilities to traditional lands (caring for country). For Australian Indigenous people one definition of the health of country is the physical presence of its traditional owners [4]. However the funding currently available is a fraction of that required for adequate management: traditional lands make up over 20% of the Australian continent (about 1.5 million square kilometres: three times the size of Spain), most of which is ecologically intact desert and tropical savannah.

Unlike with formal government-owned protected areas, funding for management of private Indigenous-owned land has not yet been integrated into mainstream Australian policies even though the environmental services provided by these lands have wider benefit for society. PES has become the economic instrument most commonly used to recognise the value of such services, and to set the right incentives for their provision [5]. Most existing successful PES schemes are in developing countries [6] where they have the potential co-benefit of lifting poor people out of poverty [1], [2]. PES could do the same for Australian Indigenous people. However few of the PES-like schemes in Australia currently involve Indigenous people, although their potential for improving social and welfare conditions in remote Indigenous communities has been recognised [7], [8]. One of the main obstacles is that most Indigenous people have group rather than individual land ownership and hence cannot sell their services as private goods [8], such as in bidding auctions. Also cultivation of Indigenous land for commercial production is rare, so there is no reason to use conservation money to set land aside. Thus, in economic terms, opportunity costs for conserving land are very low. Nevertheless there are numerous environmental problems on Indigenous-held lands that require active management if the land is to continue to provide biodiversity conservation and other environmental services [7].

The Millennium Ecosystem Assessment [9] and The Economics of Ecosystems and Biodiversity (TEEB) study [10] highlight the need to assess the value of such environmental services and their effect on human welfare [11]. Progress in this area has been impeded, however, by the lack of a standardised classification of which services to evaluate and how (e.g. [12], [13]), partly because it is often difficult to measure the biophysical outcome of services provided within a reasonable timeframe. Furthermore environmental services often have a public good character and many of the benefits, in particular those from regulation services (water and climate), are non-rival and non-excludable. This leads to under-estimation of service value, free-riding and under-supply, and finally exploitation and environmental damage [10]. What can be measured, however, and what is most important from an economic point of view, is the benefit these services have to a wider society ([14], p. 645). Public money should not in principle be spent on services for which the society gains no benefit and, vice versa, those who benefit from such services should pay for them [15]. Assessing the value of environmental benefits can help ensure public funds are distributed appropriately.

The data were obtained from a nation-wide survey that included a stated choice experiment [16]. The choice experiment method allows the monetary quantification of use values as well as of non-marketable non-use values that are often neglected in decision making for conservation management [17]. The applied choice experiment elicited respondents' willingness-to-pay for hypothetical Indigenous land management scenarios. With the chosen experimental design (see Materials and Methods) we were able to quantify the monetary benefits of three environmental services – biodiversity, aesthetic/recreational, carbon – as well as one social/cultural benefit that can occur for those providing the service (Table 1). Most benefits accrue to non-Indigenous Australians, who were the main target group for the survey. Worldwide there have been a few studies using non-market evaluation techniques to evaluate single services, such as biodiversity services (e.g. [18]) and only one study that employed a choice experiment to assess multiple ecosystem services [19]. No study, to the authors' knowledge, has assessed willingness-to-pay specifically for services delivered by Indigenous people.

**Table 1:** Attributes and levels used in a choice experiment to determine Australians willingness-to-pay for Indigenous land management.

Attribute	Levels for “Indigenous land management” scenarios	Levels for “Maintaining current management” scenario (SQ^1^)	Service/Benefit
1) Health of native animal and plant communities	- Improving- Stable	- Stable- Deteriorating	Biodiversity
2) Attractiveness for recreational use	- Good- Fair	- Poor- Fair	Aesthetic/Recreational
3) Benefits for Indigenous people	- Better health- Better transfer of Indigenous knowledge- Less dependency on the government	- No additional benefits	Social/Cultural
4) Greenhouse gas emissions	- Reducing- Stable	- Increasing- Stable	Carbon
5) Annual contribution into a conservation fund (AUS$)	$25, $50, $100, $200, $300	$0	

**Figure 1:**
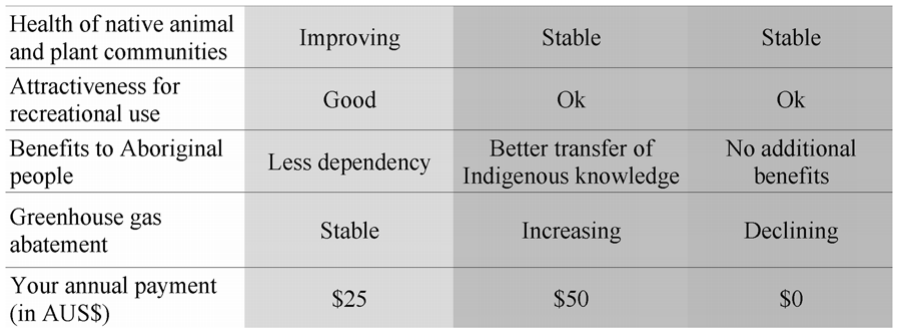
Example of a choice card used to test the willingness to pay for Indigenous provision of environmental services in northern Australia. The choice cards represented hypothetical scenarios of potential benefits from Indigenous land management. Respondents were presented with six cards, each showing different combinations of the levels. Each choice card provided three scenarios (represented by the three columns) characterised by five attributes (represented by the five rows). The column on the right always represented a situation in which current land management is maintained. The attribute in the last row represented the costs of a scenario while the other four attributes represented environmental services provided by Indigenous people. By choosing their preferred scenario each time, respondents made trade-offs between the attributes/environmental services and therefore indirectly stated their willingness-to-pay for each environmental service.

Each of the four attributes represented an environmental service/benefit. The attributes had different levels, some characterising potential Indigenous land management scenarios and some characterising a scenario resembling the current management. The levels were combined into choice cards (see Figure 1). The fifth attribute indicated the costs of the scenarios which the respondent would hypothetically be willing to contribute into an Indigenous land management conservation fund out of which Indigenous people would be paid to provide the services mentioned in the scenarios.

1 SQ: *Status quo*.

## Results

### Response rate

A total of 927 responses to the questionnaire were used in the final data analysis: 535 (42%) from the online survey and 392 (58%) from the mail survey. This represents an overall response rate of about 20% following which some responses were dismissed from the final dataset because respondents had either not attended to the choice experiment at all, had stated that they did not understood the experiment and therefore always chose not to pay, or they self-rated their degree of understanding at less than 4 (on a scale from 1 to 10 with 10 = having understood perfectly).

### Respondents' characteristics

The average age of all respondents was 51 (SD: 15; range 17–90); 53% were female. Only 2.2% of the respondents were Indigenous, slightly lower than the national average of 2.6% [20]. Seventeen percent of respondents lived in northern Australia. Twelve percent of respondents were not at all interested in Australian Indigenous culture and traditions, 52% were a little bit, 27% quite interested and 7% were fascinated by them.

### Values of environmental benefits

The best model (see Materials and Methods) included data on respondents' socio-economic background (Table 2), which explained some of the variation in preferences among respondents. As expected, respondents chose the cheapest scenario, all else being equal, but preferences varied greatly among respondents for all attributes except “improved health of native animals and plants”. Respondents preferred scenarios that ameliorated “deteriorating health of native animal and plants” and “poor recreational attractiveness”. The only benefit for Indigenous people that was significantly favoured, and so kept in the final model, was “less dependency”. “Better transfer of Indigenous knowledge” and “better health” for those providing the services were not significant in respondents' choices. Respondents felt that they would be substantially worse off from “deteriorating health of native animals and plants” (−$256) while they were willing to pay on average $80 for attractive recreational conditions, $79 for declining greenhouse gas emissions and $24 to improve environmental conditions (Table 2). Females were less likely than males to choose not to make a contribution to one of the Indigenous land management scenarios, a result found in other evaluation studies [21]. The probability of respondents choosing to pay for one of the two Indigenous land management scenarios varied inversely with age and increased with level of interest in Indigenous culture. Respondents who have lived away from northern Australia were more likely to choose to pay for one of the Indigenous land management scenarios.

**Table 2:** Results of a choice experiment to determine the willingness of Australian people to pay for Indigenous people to manage land and generate environmental services.

	Model parameters		Welfare estimates^1^ ($)	
*Variable*	*Coefficient*	*SD*	*Mean*	*CI*
Deteriorating health of native animals and plants	−2.61***	7.99***	−256	−803–287
Improving health of native animals and plants	0.22***	1.20***	24	−59–103
Low attractiveness for recreation	−0.81***	3.51***	−75	−317–157
High attractiveness for recreation	0.79***	0.85***	80	22–137
Less dependency on government for Indigenous people	−0.51***	0.59***	−50	−90–−11
Declining GHG^2^ emissions	0.77***	1.09***	79	4–151
Increasing GHG emissions	0.42***	0.29	42	23–62
Costs	−0.01***	0.01***		
Constant for SQ^3^	−1.07***			
SQ * North Australia	1.67***			
SQ * Female	−0.43***			
SQ * Age	0.04***			
SQ * Interest in Indigenous culture	−1.21***			
*Model fit:*				
Log-likelihood	−5005.14			
Pseudo R-squared	0.29			
Number of observations	6437			
Number of respondents	927			
Halton draws	200			

The standard deviations (SD) are given for those attributes which were set as random in the model. The interaction terms of the *status quo* (SQ) scenario and socio-economic parameters were set as non-random. The welfare estimates provided a monetary estimate of the benefits/disbenefits people believed they will receive from the services.

***, **, * Significance at 1%, 5%, 10% level.

1 The estimates are in AUS$ which is almost equal to the US$ (May 2011: 1US$ = 0.95 AUS$).

2 GHG: Greenhouse Gas.

3 SQ: *Status quo*.

Thirty percent of respondents chose not to contribute any money on all seven choice cards, more than half (56%) because they thought that the concept of paying Indigenous people to provide environmental services does not work, while 36% considered paying for these services to be solely a government responsibility. Other reasons included the perception that much money is already donated to environmental causes (17%), a general refusal to donate (6%) and a simple lack of money although respondents would have liked to be able to pay (3%).

### Preferred activities and aggregated values

From the four activities, which can feasibly be carried out by Indigenous people, most respondents chose feral animal control (37%) over coastal surveillance (20%), weed control (18%) and fire management (14%). Some respondents (6%) did not select any of the four activities but commented that all four are equally important and need to be carried out simultaneously. All four activities are complementary and provide, probably to a different but at the moment unknown extent, biodiversity, carbon and recreational benefits to the broader society. To assess the benefit of one individual activity, we multiplied the sum of the three environmental mean values derived from the choice experiment by the number of people who might pay and multiplied that by the percentage of respondents who selected the activity as most important. The total economic value the average respondent would receive from any of the four activities was $183 ($24 for improved biodiversity; $80 for improved recreational attractiveness; $79 for declining greenhouse gas emissions – Table 2).

Although the aggregation of values to the wider society has its limitations [22], [23], it has to be done if benefit values are to be compared with the costs [23], in this case those needed to implement a PES program for Indigenous-held land in northern Australia. If the average WTP across the sampled population were levied as a tax (see Materials and Methods) about $2.0b per year would be available with $878m (lower bound, assuming 70% of non-respondents had zero WTP) to $1.4b (upper bound, non-respondents ignored) per year being available from a voluntary scheme. If this money was allocated to different activities according to peoples' preferences the following money would be available for the following activities to be carried out by Indigenous people: feral animal control: $745m (as a tax)/$325m–$521m (voluntary payments); coastal surveillance: $403m/$176m–$282m; weed control $362m/$158m–$253m; and fire management $282m/$123m–$197m.

## Discussion

Government initiatives for Indigenous land management (Working on Country, Indigenous Rangers, Indigenous Protected Areas) currently provide about $40 million per year [24]. This budget is only secure until 2013 but knowing that the beneficiaries of the environmental services emerging from this program are willing to pay up to 50 times this amount is an argument for its continuity. The difficulty is translating this WTP into increased funds for Indigenous land management. A nation-wide tax scheme would force people to pay unwillingly. Ironically resistance to such a tax is likely to be greatest among the people who live in or visit northern Australia, i.e. those who potentially benefit most from at least some of the services, such as improved recreational conditions. This study showed that these people were less likely to support the funding of Indigenous people to provide environmental services than people living in distant southern Australia, which contrasts with studies elsewhere showing an inverse relationship between distance and the amount people are willing to pay for such services (e.g. [25]). This negative attitude among northern residents and visitors also mitigates against raising annual fees for “consuming” recreational and biodiversity benefits in northern Australia. Although fees are indeed levied in some protected areas, where visitors are required to purchase entry permits, the amounts collected are well below the costs of expanding Indigenous land management.

Instead the greatest value from Indigenous land management is to those in southern Australian who have not benefited directly from the north Australian environment, and may never do so. For this sector voluntary WTP may be translated into actual benefits for service providers most equitably and efficiently through agreements between traditional landowners and private companies. In particular northern Australia could become very attractive in the future to those wishing to invest in schemes for reducing emissions of greenhouse gases, for which examples already exist [26].

Another potential source of land management funding is savings that arise from improvements in health among Indigenous people that occur when they spend more time caring for country. For example, substantial savings are realised from reduction in the incidence of diabetes, heart disease and renal failure as engagement with land management increases [27]. Given the health benefits, some health funds could be redirected to Indigenous land management, reducing the amount needed from donations to conservation schemes [7]. However the respondents were more enthusiastic about a reduced dependence on welfare among Indigenous people than the health benefits that might accrue to them. The most likely reason for this is that the knowledge of such benefits is not yet widely known. The study cited [27] is one of just a handful of recent studies formally linking health gains to caring for country, and the first to quantify economic savings.

While our study focused on the value of the services provided to non-Indigenous people, more research is needed to investigate the willingness of Indigenous people to participate and how they would like to be compensated for their work. While the needs of those who would pay (mostly non-Indigenous people) eventually have to match the services that are provided, the capacity and willingness of Indigenous people to provide the services is critical to any PES scheme's success. Given that Indigenous people have demonstrated a determination to meeting cultural obligations to their country despite the disincentive of poor services in remote areas [28], the strict assumptions of conventional PES schemes as conditional, well defined and voluntary [29] may need to be reconsidered. Hence, evaluating environmental services from a benefit-based approach, as we have done in this study, may be more appropriate than a rigid contractual link between the services to be provided and the manner in which this is to occur. Taking into account Indigenous understanding of their environment is particularly important. Although we show that respondents have preferences for some activities they would like to have carried out for their money, such as pest control, Indigenous people will often know best how to achieve the benefits from their services. Despite these caveats this research has demonstrated that Australians may be willing to pay Indigenous people far more than they currently do to prevent deterioration of the environment in northern Australia.

## Materials and Methods

### Data collection and ethics statement

We used a structured questionnaire which consisted of three parts: 1) questions related to respondents' socio-economic status including attitudinal questions, 2) the choice experiment including prior information on activities that Indigenous people commonly carry out on their land and their potential benefits, and 3) follow-up questions from the choice experiment. The questionnaires were distributed to 4,600 people across Australia; 3,000 questionnaires were mailed to mostly urban addresses in southern Australia and 1,000 to addresses across northern Australia which is where most Indigenous ranger employment is occurring. All mail-out addresses were randomly selected from the telephone directory. An online version of the same questionnaire was distributed to 560 respondents by an Online market company. Online recipients were randomly approached by the Online market company within predefined strata, ensuring that the sample population represented an equal gender ratio, a full range of age categories and all main urban regions in Australia. Ethics approval for the survey was obtained from the Charles Darwin University Human Research Ethics Committee (EC00154). A letter in plain English language explaining the purpose of the survey, how to complete it and stating that the survey was voluntary and anonymous accompanied each questionnaire. By answering and returning the questionnaire respondents were assumed to have given consent.

### Choice experimental design

In a choice experiment respondents are asked to choose between alternative scenarios, defined in terms of their attributes. In our experimental design we described the attributes in terms of benefits from environmental services from Indigenous land management (Table 1). These benefits and the levels were selected after interviews with key informants and a pre-test period. The benefits have various levels that vary from scenario to scenario according to specific design rules. In this study there were always three scenarios to choose from (Figure 1) on one choice card. First of all these were two Indigenous land management scenarios which would improve or stabilise environmental conditions and benefit the Indigenous people providing the services. These two scenarios came with a putative personal cost to the respondents. Including this cost attribute, described as an annual one-off contribution per household into a conservation fund from which Indigenous people would be paid to provide the environmental services, enabled the calculation of welfare estimates. The third scenario (“Maintain current management”, the *status-quo* scenario) described as a situation in which no additional money would be available for employing Indigenous people to work on country with a probable result that environmental conditions would at best stay stable, and would probably deteriorate.

The specified number of attributes and their levels (Table 1) allowed many potential combinations into scenarios and choice cards. Since it was impractical to use them all, we applied a Bayesian approach to obtain a statistically efficient design with 30 choice cards, using the software Ngene. Efficient designs aim to provide data that generates parameter estimates with standard errors as small as possible [30]. Efficient designs, now used commonly for choosing the best experimental design [31], will outperform other commonly used designs if any prior information on the parameters is available, even if the priors are mis-specified [30]. We derived prior parameter information from a pilot phase with 30 respondents which we used to create the final design. The Bayesian D-error of the final design was 0.018.

From the 30 choice cards only 21 were used after behaviourally unrealistic cards and those requiring no trade-offs were removed. The remaining 21 cards were randomly blocked into three versions and each respondent was given seven choice cards in a questionnaire. Each version was used the same number of times. In addition, we accounted for a potential left-right bias by alternating the order of appearance of the two Indigenous land management scenarios on the choice cards.

### Data analysis

Data was analysed using the software package Limdep Nlogit 4.0 and all attribute variables but the cost attribute were dummy-coded (0/1). Lancaster' s characteristics theory of value [32] and random utility theory (RUT) [33], in which respondents make discrete choices from a set of scenarios which reflect their probable behaviour, underpin the choice experiment approach. RUT states that respondent **j**'s utility for scenario **i** consists of two parts, an observable and deterministic part **X_ji_b** and an unobservable part **e_ji_** [34]:
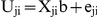
where **X_ji_** is a matrix of scenario **i**'s attributes and **b** is a vector of unknown coefficients associated with these attributes. **e_ji_** is the *iid* (independently and identically distributed) maximum extreme value type I distribution error term [16], [35]. According to RUT, respondents will chose the scenario out of a choice card that gives them the highest utility. Based on this assumption, the conditional logit model can be formed [16]:
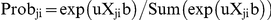
The scale vector **u** is assumed to equal one (implying constant error variance) so that the model becomes deterministic and the **b**'s can be identified. We applied random parameter logit (RPL) models because of their flexibility with respect to *iid* error terms, to address unobserved preference heterogeneity [36] and to take full advantage of panel data. We compared different models using log-likelihood ratio tests.

The welfare estimation (here the benefit values) of the four individual services can then be carried out by calculating the ratio −b_j_/b_cost_, where **b_j_** is the coefficient for an attribute **j** in the choice card and **b_cost_** is the attribute associated with the money respondents would pay for the chosen scenarios (here annual contribution into a conservation fund for Indigenous land management). These ratios can be calculated for each attribute level while holding the other attributes at the same levels. For RPL models the welfare estimates are approximated via simulations [16], [37]. Using the parametric bootstrapping technique [38] we estimated a distribution of 10,000 observations for each welfare estimate by drawing from a multivariate normal distribution parameterised with the coefficient and standard deviation obtained from the models. This method also provides the 95% confidence intervals for each welfare estimate.

### Aggregation of values

To aggregate we considered two approaches for collecting the money needed to pay for Indigenous provision of environmental services: a national tax paid by every employed Australian (11 m people [20]) and a voluntary payment scheme. For the voluntary scheme we calculated a lower and upper bound. For the upper bound we assumed that 70% of taxpayers (about 7.7 m people), based on those willing to pay in this survey, are willing to pay the amounts they specified in the survey. In this case we did not consider those respondents who did not send back the questionnaire or did not fill out the online survey. For the lower bound we followed the method proposed by Morrison [22] and assumed that the proportion of non-respondents who did not send back the questionnaire had the same WTP as the respondents.
